# Hurricane Exposure and Risk of Long-Term Cardiovascular Disease Outcomes

**DOI:** 10.1001/jamanetworkopen.2025.30335

**Published:** 2025-09-03

**Authors:** Arnab K. Ghosh, Orysya Soroka, Monika Safford, Martin F. Shapiro, Fei Wang, Glen D. Johnson, Yasin Civelek, Charles DiMaggio, David Abramson

**Affiliations:** 1 Department of Medicine, Weill Cornell Medicine, New York, New York; 2 Department of Population Health Sciences, Weill Cornell Medicine, New York, New York; 3 Department of Environmental, Occupational and Geospatial Health Sciences, City University of New York Graduate School of Public Health and Health Policy, New York, New York; 4 Cornell Health Policy Center, New York, New York; 5 Department of Surgery, New York University School of Medicine, New York, New York; 6 School of Global Public Health, New York University, New York, New York

## Abstract

**Importance:**

Hurricanes are associated with increased cardiovascular disease (CVD) risk, yet little is known about whether these risks extend into the long term and for how long.

**Objective:**

To examine the association between hurricane-related flooding and CVD risk up to 5 years after landfall.

**Design, Setting, and Participants:**

This cohort study included a 20% national sample of continuously enrolled Medicare fee-for-service beneficiaries from New Jersey, New York City, and Connecticut from January 1, 2010, to December 31, 2017. Data were analyzed from December 14, 2023, to June 20, 2025.

**Exposure:**

Residence in zip code tabulation areas (ZCTAs) impacted by flooding from Hurricane Sandy throughout the study period.

**Main Outcomes and Measures:**

The primary outcome was the rate of ZCTA-level adjusted CVD events (including myocardial infarction [MI], heart failure [HF], and stroke), defined per 1000 beneficiary-years, with associated 95% bayesian credible intervals (bCrIs). Secondary outcomes included the rate of events for each CVD subtype (MI, HF, and stroke).

**Results:**

In the matched cohort, 121 395 beneficiaries resided in 690 ZCTAs, of which 441 (63.9%) flooded. In nonflooded vs flooded ZCTAs, mean (SD) age (74.2 [1.4] vs 74.1 [1.2] years; *P* = .16), proportion of female beneficiaries (61.4% [8.4%] vs 61.3% [6.6%]; *P* = .89), and proportion of White beneficiaries (74.3% [28.7%] vs 76.7% [26.8%]; *P* = .27) were similar, but ZCTA-level median income ($81 168 [$33 410] vs $69 650 [$27 594]; *P* < .001) and median National Area Deprivation Index rank (17.1 [IQR, 10.2-27.6] vs 21.0 [IQR, 10.9-32.5]; *P* = .02) differed; prevalence of CVD and CVD subtypes was similar at baseline. Flooding was associated with an increase in adjusted 5-year CVD risk post landfall (relative risk, 1.05; 95% bCrI, 1.01-1.08) and HF rates overall (relative risk, 1.03; 95% bCrI, 1.00-1.08). No significant difference was found in rates of MI or stroke in adjusted analyses.

**Conclusions and Relevance:**

In this cohort study of Medicare fee-for-service beneficiaries, hurricane-related flooding was associated with increases in CVD event rates as long as 5 years after landfall and increased HF rates in New Jersey. These findings highlight the importance of place-based vulnerability from hurricane exposure to mitigate longer-term CVD risk and the need to consider long-term outcomes in hurricane mitigation efforts.

## Key Points

**Question:**

What is the long-term association of hurricane-related flooding with cardiovascular disease (CVD) risk?

**Findings:**

In this cohort study of 121 395 continuously enrolled Medicare fee-for-service beneficiaries residing in New Jersey, New York City, and Connecticut, 5-year CVD event rates were higher for zip codes exposed to flooding from Hurricane Sandy than nonflooded zip codes, with the greatest risk noted approximately 3 years after landfall for heart failure events in New Jersey.

**Meaning:**

These findings suggest that hurricane flooding is associated with long-term CVD risk up to 5 years after landfall.

## Teaser

This cohort study examines the association between hurricane exposure and the long-term risk of cardiovascular disease events after Hurricane Sandy in 2012 among Medicare fee-for-service beneficiaries residing in New Jersey, New York City, and Connecticut.

## Introduction

Driven by anthropogenic climate change, hurricanes, cyclones, and severe storms are increasing in strength and frequency, leading to widespread destruction in impacted areas.^1,2^ Furthermore, there is growing evidence that hurricane intensification (the rapid escalation in hurricane categorization) is taking place,^3^ leading to shorter periods for adequate disaster preparedness and response planning by emergency management authorities, communities, and individuals.

Devastation wrought by hurricanes has resulted in adverse health-related impacts in the weeks and months after a hurricane’s landfall.^4^ Notably, exposure to hurricanes has been associated with increases in cardiovascular disease (CVD) events, including mortality from acute myocardial infarction (AMI), heart failure (HF), and stroke in the months following.^5^ Furthermore, in the short term and likely due to the direct exposure to a hurricane’s impact, older adults exposed to a hurricane’s path are at increased risk of CVD-related hospitalization and emergency department visits, as well as exacerbations of other chronic conditions.^6,7,8^ Moreover, adverse health outcomes related to hurricane impacts have been well characterized in patients with diabetes^9^ and hypertension^6^ as well as among racial and ethnic minoritized populations, all of whom are at increased risk of CVD events.

While the short-term association of hurricanes with CVD outcomes have been well described,^10,11^ few studies have rigorously examined the association of hurricane exposure with longer-term CVD risk.^4,12,13^ The long-term impacts of hurricanes on older adults is important to understand for 3 reasons. First, demographic shifts toward an older population combined with aging in place^14^ and the importance of place-based attachment^15^ are likely to increase the risk of hurricane exposure and associated long-term sequelae^16^ (eg, health care disruption,^17,18^ disaster-related gentrification,^19,20^ changes to socioeconomic environments, and population shifts^21^), all of which may increase CVD risk. Second, such disruption is likely to be amplified because hurricane-related impacts are more likely to extend into new regions that lack knowledge and experience in emergency management before and after hurricanes (eg, the impact of Hurricane Helene in western North Carolina in 2024) or are more likely to repeatedly strike the same areas (eg, the southeastern US). Third, current disaster management strategies, including the Federal Emergency Management Agency National Disaster Recovery Framework,^22^ emphasize short-term emergency health care access in the first months after the event. As a result, policies that seek to examine and mitigate the likely long-term health impacts of hurricanes are limited despite a likely need.

To address this problem, we used rigorous spatiotemporal econometric methods (difference-in-differences and event study specifications)^23^ to examine the association between hurricane exposure and risk of CVD events into the longer term after Hurricane Sandy in 2012 among Medicare fee-for-service (FFS) beneficiaries residing in New Jersey, New York City (NYC), and Connecticut, the 3 regions most impacted by flooding. We chose Hurricane Sandy as an example because of its widespread devastation to a highly populated area of the US.^24,25^ It also presented an opportunity to examine long-term CVD outcomes before the advent of the COVID-19 pandemic. We chose to examine this empirical question in a socioecological framework (ie, using zip code tabulation area [ZCTA] as our unit of analysis) because hurricane exposure is spatially distributed and the socioecological framework is commonly used by public health–related disaster preparedness.^26^

## Methods

### Study Design, Sample, and Setting

In this cohort study, we conducted ZCTA-level spatiotemporal difference-in-differences and event study analyses to compare adjusted CVD event rates in flood-impacted ZCTAs with those in nonimpacted ZCTAs across NYC, New Jersey, and Connecticut from January 1, 2010, to December 31, 2017, 5 years after Hurricane Sandy’s landfall. Five years after the event was chosen based on the previous scoping review by some of us^4^ of studies that examined the long-term association of hurricanes with CVD risk, which found the most rigorous studies address long-term risk at 1 year after hurricane landfall.^9^ The study protocol was approved by the Weill Cornell Medicine institutional review board on August 9, 2023, and informed consent was because of use of deidentified data. This report followed the Strengthening the Reporting of Observational Studies in Epidemiology (STROBE) reporting guideline for cohort studies.

We used a 20% nationally representative sample of Medicare FFS beneficiaries and their associated claims. The study cohort was defined as beneficiaries who were continuously enrolled in Medicare Parts A and B and were 65 years or older from 2010 onward. Beneficiaries were also required to remain in the same ZCTA (data taken from the Medicare Beneficiary Summary File) from 2010 onward (eFigure 1 in Supplement 1) to ensure that, for the purposes of our analytical strategy, the stable unit treatment assumption of difference-in-differences analysis was met. We chose to undertake this analysis at the ZCTA level, as this is the smallest administrative unit for Medicare beneficiaries available from the yearly Medicare Beneficiary Summary Files.

### Exposure

Our exposure variable was the presence or absence of hurricane-related flooding due to the storm surge for each ZCTA, consistent with other hurricane-related studies.^6,27^ We obtained flooding data from the US Geological Survey highwater marks mapping from October 2012. These flood maps represent the actual flooding that occurred during Hurricane Sandy. To examine whether ZCTAs were impacted by floods, we mapped these flood data onto ZCTA-level shapefiles. To ensure that we were making geographically appropriate comparisons between flood-impacted and nonimpacted ZCTAs, we limited our study sample of ZCTAs to only those within a 10-mile radius of the boundary of the flooded ZCTA.

### Outcomes

The primary outcome consisted of rates of CVD events, which were calculated for each ZCTA from Medicare FFS Part A and B files using validated claims-based algorithms for HF, stroke, and AMI (eTable 1 in Supplement 1).^28,29,30^ Within each year quarter, events were calculated for each Medicare FFS beneficiary, aggregated at the ZCTA level, and converted into rates by adjusting for the period during which the beneficiary remained enrolled in Medicare (per 1000 beneficiary-years).

### Covariates

Covariate selection was informed by the socioecological model of disaster recovery^31^ and a conceptual model that also served as a directed acyclic graph (eFigure 2 in Supplement 1). Two types of time-varying covariates were included in the model. The first was calculated directly from the Medicare FFS cohort at the ZCTA level (mean age, proportion of female beneficiaries, and self-reported race and ethnicity). To address the role of confounding by comorbidities, for each beneficiary a yearly adjusted Charlson Comorbidity Index score was calculated (after removing CVD, including coronary heart disease, stroke, and HF), aggregated and with the mean calculated for each ZCTA.

American Community Survey 5-year estimates provided disaster-specific area-level covariates at the yearly level.^32,33,34,35,36,37^ These included yearly ZCTA-level estimates of residents older than 65 years, median income, proportion of White residents, proportion of renter-occupied households, and proportion of overcrowded households, defined as the estimated number of housing units with more than 1 occupant per room divided by the number of occupied housing units and expressed as a percentage within each ZCTA. Adding data for other race and ethnicity does not provide additional information to the model. To address overall disadvantage at the ZCTA level that allowed comparison across the 3 regions, we also included the non–time-varying nationally ranked Area Deprivation Index^38^ (using data from 2011-2015) as a covariate.

### Statistical Analysis

Baseline characteristics of the cohort at the ZCTA level were summarized using means for continuous values and proportions for categorical values by flood-impacted vs non–flood-impacted ZCTAs. Differences were compared using *t* tests and χ^2^ tests where appropriate. We performed propensity score matching on sociodemographic and clinical covariates, showing good balance using the standardized mean difference.

We then mapped ZCTA-level CVD event rates for each year to determine the need for building a model that accounts for spatial and/or temporal correlation. We measured spatial autocorrelation with the Moran *I* statistic for each year quarter for the CVD outcome, determining that spatial clustering existed and varied over time (eTable 2 in Supplement 1).

To account for this spatiotemporal autocorrelation, we used a bayesian model that accounted for spatial dependence on adjacent ZCTAs through a conditional autoregressive random effect in addition to the unstructured variance term (the Besag-York-Mollié convolution model^39^) and an additional temporal random effect modeled as a random walk, which assumed that the event rate in the ZCTA at time *t* in a specific year quarter was equal to the rate at time *t* − 1 plus random noise that was normally distributed with mean 0. The integrated nested Laplace approximation method was applied, yielding posterior distributions of all stochastic nodes in the model.

Using this model specification, we then estimated the association between hurricane flooding and long-term CVD event rates at the ZCTA level using a negative binomially distributed difference-in-differences approach with matching. Using all the covariates in our model (ie, mean age of Medicare FFS beneficiaries in the ZCTA, proportion of female Medicare FFS beneficiaries, mean adjusted Charlson Comorbidity Index score, Area Deprivation Index, proportion of individuals in the ZCTA 65 years or older, proportion of all renter-occupied households and overcrowded households in the ZCTA, proportion of residents who belonged to racial and ethnic minority groups, and the state in which the ZCTA was located), we used 1:1, 2:1, and 3:1 nearest-neighbor matching with replacement on the prehurricane trends to address the differences in observed characteristics between flood-impacted and nonimpacted ZCTAs. We tested the balance of matching using standardized mean difference,^40^ finding that 3:1 nearest-neighbor matching with replacement minimized the standardized mean difference across the matched covariates (eFigure 3 in Supplement 1). After matching, we estimated a multivariable regression model in which a ZCTA’s exposure to hurricane flooding was a binary indicator and interacted against the time in the year after Hurricane Sandy’s landfall (which took place in October 2012). This analysis was performed for the primary outcome of interest (CVD event rates) and secondary outcomes (CVD subtypes [AMI, HF, and stroke]). In addition to the covariates listed above and spatial and temporal random effects, all regression models were adjusted for a time-year intercept (ie, year fixed effects). We undertook subgroup analyses for each region (NYC, New Jersey, and Connecticut).

To allow the associations between hurricane flooding and CVD event rates to vary over time within the difference-in-differences framework, we also used an event study specification.^23^ We estimated the multivariable regression model where the independent variables of interest were a series of binary indicators denoting each year quarter before and after Hurricane Sandy’s landfall. Using this specification allowed us to compare CVD event rates for each year quarter increment before and after the hurricane’s landfall. It also enabled us to test the parallel trends assumption of the difference-in-differences framework (the key identifying assumption for causal inference) and examine how long after the hurricane’s landfall the rates remained heightened. Details of the estimating equation for both specifications can be found in eFigure 4 in Supplement 1.

For all models, we compared the posterior mean of the difference-in-differences estimator and associated 2.5th and 97.5th quantiles of the mean to describe 95% bayesian credible intervals (95% bCrIs). All analyses were conducted in R, version 4.5.0 (R Program for Statistical Computing), and ArcGIS Pro, version 3.4.3 (ESRI), and data were analyzed from December 14, 2023, to June 20, 2025.

We conducted several sensitivity analyses. First, we assessed different matching strategies (1:1 and 2:1 replacement) and examined matching diagnostics and application of these matching strategies to the primary analysis (eFigure 3 in Supplement 1). Second, to address concern that some ZCTAs had few Medicare beneficiaries, all models were run removing ZCTAs with fewer than 50 and fewer than 100 beneficiaries, and the estimators were compared. Third, we also ran the analyses using generalized linear models without the bayesian framework (eTable 3 in Supplement 1).

## Results

### Baseline Characteristics

**Figure 1.**
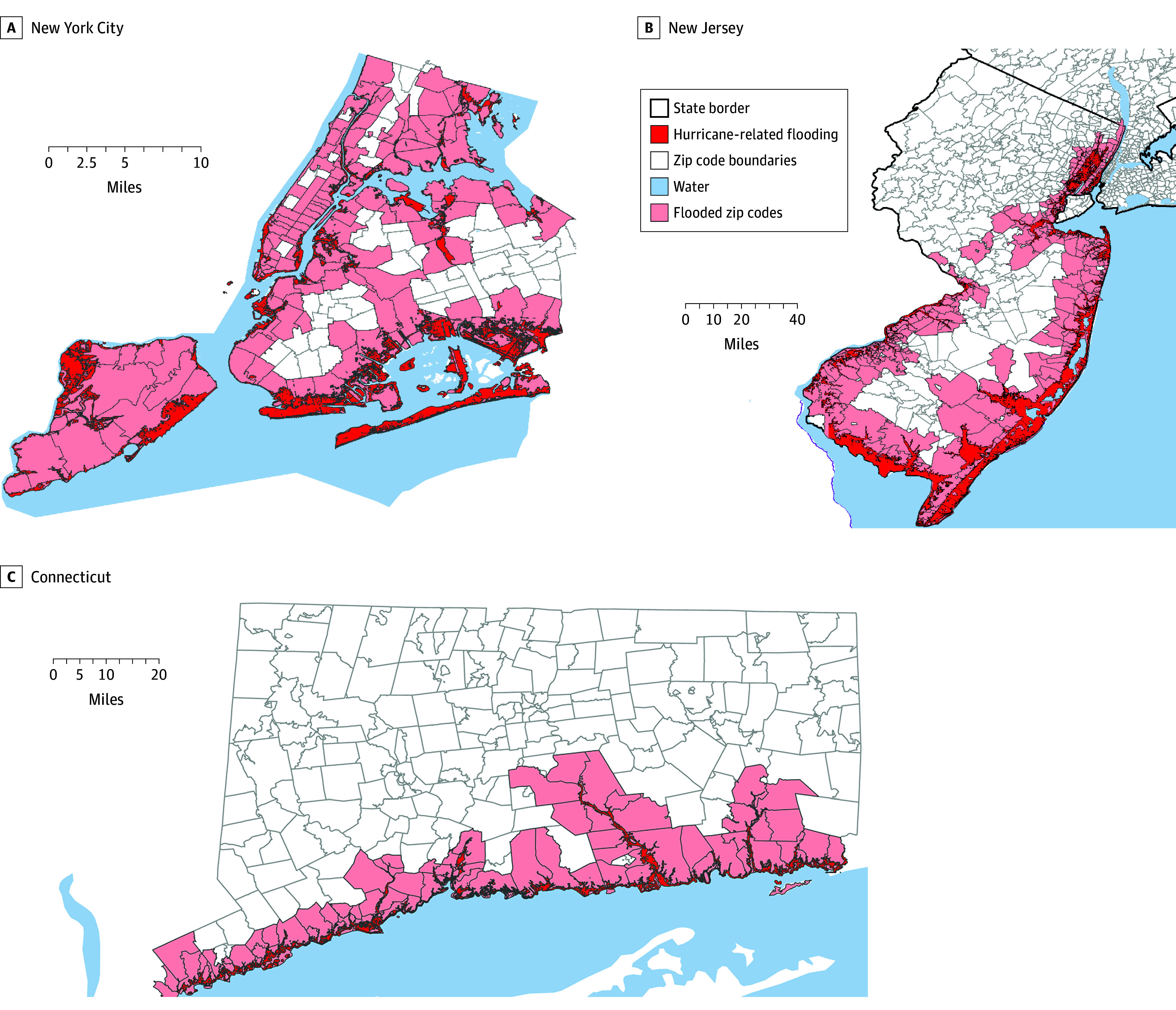
Zip Code Tabulation Areas Impacted by Hurricane-Related Flooding Across the 3 Study Regions Flood impacts were determined from US Geological Survey highwater marks mapping from October 2012. Maps consist of only zip code tabulation areas within a 10-mile radius of the flooded region.

Figure 1 graphically describes the spatial distribution of flooded ZCTAs. Across all the study regions, hurricane-related flooding took place along the coastlines and also topographically lower lying areas inland. Table 1 describes the baseline characteristics of ZCTAs stratified by exposure to hurricane flooding before and after matching at the beginning of the study period in 2010. Before matching, there were 121 599 beneficiaries in the cohort. Four hundred and forty-four of the 695 ZCTAs in NYC, New Jersey, and Connecticut (63.9%) experienced hurricane-related flooding. Mean (SD) age of the Medicare FFS beneficiaries in nonflooded (74.2 [1.4] years) and flooded (74.1 [1.2]) ZCTAs was comparable (*P* = .18), as were the mean (SD) proportion of female (nonflooded, 61.5% [8.4%]; flooded 61.3%, [6.6%]; *P* = .75) and male (nonflooded, 38.5% [8.4%]; flooded, 38.7% [6.6%]; *P* = .75) beneficiaries and mean (SD) proportion of self-reported White beneficiaries (nonflooded, 74.0% [29.0%]; flooded, 76.7% [36.8%]; *P* = .21). The mean (SD) proportions of reported Black (nonflooded, 14.4%, [25.2%]; flooded, 9.9% [17.6%]; *P* = .01) and Hispanic (nonflooded, 5.8% [8.4%]; flooded, 8.6% [14.0%]; *P* = .01) beneficiaries were significantly different.

**Table 1.** Baseline Characteristics of ZCTAs Stratified by Flood Impact

Characteristic	Unmatched cohort^a^			Matched cohort^b^		
	Nonimpacted	Flood impacted	*P* value	Nonimpacted	Flood impacted	*P* value
No. of ZCTAs	251	444	NA	249	441	NA
No. of Medicare FFS beneficiaries	39 818	81 781	NA	39 645	81 750	NA
Beneficiary-level demographic and clinical characteristics						
Age, mean (SD), y^c^	74.2 (1.4)	74.1 (1.2)	.18	74.2 (1.4)	74.1 (1.2)	.16
Sex, mean (SD), %						
Female	61.5 (8.4)	61.3 (6.6)	.75	61.4 (8.4)	61.3 (6.6)	.89
Male	38.5 (8.4)	38.7 (6.6)	.75	38.6 (8.4)	38.7 (6.6)	.89
Race and ethnicity, mean (SD), %						
Asian	3.8 (5.3)	3.1 (5.4)	.11	3.8 (5.3)	3.1 (5.4)	.10
Black	14.4 (25.2)	9.9 (17.6)	.01	14.1 (24.8)	10.0 (17.6)	.01
Hispanic	5.8 (8.4)	8.6 (14.0)	.01	5.8 (8.4)	8.6 (14.0)	.01
White	74.0 (29.0)	76.7 (26.8)	.21	74.3 (28.7)	76.7 (26.8)	.27
No. of chronic conditions, mean (SD)	5.5 (0.8)	5.6 (0.8)	.09	5.5 (0.8)	5.6 (0.8)	.08
ZCTA-level socioeconomic characteristics^d^						
Age ≥65 y, mean (SD), %	13.9 (7.1)	14.2 (6.6)	.44	14.0 (7.1)	14.4 (6.4)	.43
White residents, mean (SD), %	66.5 (28.5)	70.2 (24.7)	.08	66.7 (28.3)	70.2 (24.8)	.09
Income, median (SD), 2010 USD	81 410 (33 618)	69 721 (27 612)	<.001	81 168 (33 410)	69 650 (27 594)	<.001
Renter-occupied households, mean (SD), %	31.7 (24.5)	40.3 (26.0)	<.001	31.8 (24.6)	40.0 (25.9)	<.001
Overcrowded households, mean (SD), %	3.8 (6.4)	3.8 (4.7)	.92	3.8 (6.4)	3.8 (4.7)	.96
National ADI rank, median (IQR)^e^	17.0 (10.2, 27.6)	21.0 (10.6-32.4)	.03	17.1 (10.2-27.6)	21.0 (10.9-32.5)	.02
ZCTA-level prevalence statistics, mean (SD), %^f^						
CVD	24.1 (8.4)	24.0 (8.8)	.86	24.1 (8.4)	23.9 (8.8)	.86
AMI	2.0 (1.6)	2.1 (1.7)	.59	2.1 (1.6)	2.1 (1.7)	.58
Stroke	8.2 (4.8)	8.2 (4.1)	.88	8.2 (4.9)	8.2 (4.1)	.88
Congestive heart failure	18.6 (8.6)	18.8 (8.8)	.80	18.6 (8.6)	18.8 (8.8)	.79

Abbreviations: ADI, Area Deprivation Index; AMI, acute myocardial infarction; CVD, cardiovascular disease; FFS, fee for service; NA, not applicable; ZCTA, zip code tabulation area.

^a^ From 2010, the first year of the study period.

^b^ Calculated using 1:3 nearest-neighbor matching with replacement.

^c^ For each ZCTA, mean age of study participants was calculated. Then the mean age across all ZCTAs was calculated.

^d^ From the American Community Survey 2010 (5-year mean during the 2006-2010 period).

^e^ Represents the relative socioeconomic conditions of neighborhoods as a percentile of national ranking at the ZCTA level.

^f^ Calculated from the Chronic Condition Warehouse for each Medicare FFS beneficiary in 2010.

In unmatched analysis, ZCTA-level socioeconomic characteristics were similar for nonflooded and flooded ZCTAs except for median (SD) income (nonflooded, $81 410 [$33 618]; flooded, $69 721 [$27 612]; *P* < .001), mean (SD) proportion of renter-occupied households (nonflooded, 31.7% [24.5%]; flooded, 40.3% [26.0%]; *P* < .001) and median Area Deprivation Index rank (nonflooded, 17.0 [IQR, 10.2-27.6]; flooded, 21.0 [IQR, 10.6-32.4]; *P* = .03). All ZCTA-level prevalence statistics (CVD, AMI, congestive HF, and stroke) were similar between nonflooded and flooded ZCTAs.

After matching, there were 121 395 beneficiaries across 249 nonflooded ZCTAs and 441 flooded ZCTAs. In nonflooded vs flooded ZCTAs, mean (SD) age (74.2 [1.4] vs 74.1 [1.2] years; *P* = .16), proportion of female beneficiaries (61.4% [8.4%] vs 61.3% [6.6%]; *P* = .89), and proportion of White beneficiaries (74.3% [28.7%] vs 76.7% [26.8%]; *P* = .27) were similar, but ZCTA-level median income ($81 168 [$33 410] vs $69 650 [$27 594]; *P* < .001) and median national area deprivation index rank (17.1 [IQR, 10.2-27.6] vs 21.0 [IQR, 10.9-32.5]; *P* = .02) differed significantly. Although matching did not change the significant differences between baseline characteristics in 2010 of nonflooded and flooded ZCTAs, the standard mean difference was less than 0.01 across all matched covariates (eFigure 3 in Supplement 1).

### Difference-in-Differences Estimates

Table 2 describes the results of the spatiotemporal difference-in-differences models for overall CVD event rates across all study regions, within each study region, by each CVD subtype, and with associated sensitivity analyses including only ZCTAs with greater than 50 and greater than 100 Medicare FFS beneficiaries. The adjusted CVD event rates comparing the period before the hurricane’s landfall and 5 years after landfall were higher (relative risk [RR], 1.05; 95% bCrI, 1.01-1.08) in flooded ZCTAs compared with nonflooded ZCTAs. The risk was greater with the removal of ZCTAs with less than 50 beneficiaries (RR, 1.06; 95% bCrI, 1.02-1.09) and 100 beneficiaries (RR, 1.07; 95% bCrI, 1.03-1.11). In the subgroup analysis across all regions, hurricane-related flooding was associated with increased adjusted HF event rates overall (RR, 1.03; 95% bCrI, 1.00-1.08) and in ZCTAs with 50 or more beneficiaries (RR, 1.05; 95% bCrI, 1.01-1.09) and 100 or more beneficiaries (RR, 1.06; 95% bCrI, 1.02-1.10). However, among all regions, hurricane-related flooding was not associated with increased adjusted rates of AMI or stroke among all ZCTAs and after excluding ZCTAs with less than 50 or less than 100 beneficiaries.

**Table 2.** Adjusted RR in CVD Outcome Rates From 2 Years Before to 5 Years After Hurricane Sandy Landfall, Between Matched Flood-Impacted and Nonimpacted ZCTAs by Region, 2011 to 2017^a^

Outcome	Adjusted RR (95% bCrI)			
	All regions	NYC	New Jersey	Connecticut
**CVD**				
All ZCTAs	1.05 (1.01-1.08)^b^	1.04 (0.98-1.09)	1.06 (1.00-1.11)^b^	0.95 (0.83-1.08)
ZCTAs with ≥50 beneficiaries	1.06 (1.02-1.09)^b^	1.04 (0.99-1.10)	1.07 (1.01-1.12)^b^	0.99 (0.87-1.14)
ZCTAs with ≥100 beneficiaries	1.07 (1.03-1.11)^b^	1.04 (0.99-1.10)	1.08 (1.03-1.14)^b^	0.99 (0.85-1.15)
**Myocardial infarction **				
All ZCTAs	1.06 (0.93-1.20)	0.94 (0.76-1.17)	1.16 (0.99-1.35)	0.9 (0.60-1.36)
ZCTAs with ≥50 beneficiaries	1.06 (0.94-1.20)	0.95 (0.76-1.18)	1.14 (0.98-1.35)	0.95 (0.63-1.44)
ZCTAs with ≥100 beneficiaries	1.05 (0.92-1.20)	0.94 (0.74-1.18)	1.12 (0.95-1.34)	1.01 (0.63-1.60)
**Heart failure**				
All ZCTAs	1.03 (1.00-1.08)	1.04 (0.99-1.10)	1.04 (0.99-1.10)	0.92 (0.80-1.06)
ZCTAs with ≥50 beneficiaries	1.05 (1.01-1.09)^b^	1.05 (0.99-1.10)	1.10 (1.03-1.18)^b^	0.96 (0.80-1.12)
ZCTAs with ≥100 beneficiaries	1.06 (1.02-1.10)^b^	1.05 (0.99-1.11)	1.07 (1.01-1.14)^b^	0.92 (0.77-1.09)
**Stroke**				
All ZCTAs	1.06 (0.94-1.21)	0.92 (0.72-1.18)	1.08 (0.92-1.27)	1.29 (0.85-1.95)
ZCTAs with ≥50 beneficiaries	1.09 (0.96-1.24)	0.9 (0.70-1.16)	1.13 (0.96-1.34)	1.38 (0.90-2.12)
ZCTAs with ≥100 beneficiaries	1.11 (0.97-1.28)	0.92 (0.71-1.19)	1.14 (0.95-1.36)	1.57 (0.96-2.57)

Abbreviations: bCrI, bayesian credible interval; CVD; cardiovascular disease; NYC, New York City; RR, relative risk; ZCTA, zip code tabulation area.

^a^ All models used a bayesian spatiotemporal convolution model that incorporated negative-binomial distributed log-link function to model the outcome. ZCTAs were matched on prehurricane trends using a 1:1 nearest-neighbor matching strategy with replacement. All models were adjusted for ZCTA-level covariates calculated from the American Community Survey 5-year estimates (proportion of White residents, Area Deprivation Index, proportion of residents in ZCTA 65 years or older) and ZCTA-level covariates calculated from Medicare fee-for-service beneficiaries (mean age, proportion of racial and ethnic minority residents, mean adjusted Charlson Comorbidity Index score); models also included region-specific intercepts and a spatiotemporal random effect with the temporal component modeled with a random walk and the spatial random effect defined by Besag-York-Mollié model that smooths data according to neighborhood structure and also uncorrelated spatial noise.

^b^ *P* < .05.

In analyses by each region and event subtype, hurricane-related flooding was not associated with increased adjusted rates of CVD, AMI, stroke, or HF subtypes in the regions of NYC or Connecticut. However, hurricane-related flooding was associated with increased adjusted CVD events in New Jersey (RR, 1.06; 95% bCrI, 1.00-1.11), among ZCTAs with 50 or more beneficiaries (RR, 1.07; 95% bCrI, 1.01-1.12), and among those with 100 or more beneficiaries (RR, 1.08; 95% bCrI, 1.03-1.14). Among the CVD subtypes, only increased adjusted HF rates were associated with hurricane-related flooding in New Jersey, but only in ZCTAs with 50 or more beneficiaries (RR, 1.10; 95% bCrI, 1.03-1.18) and 100 or more beneficiaries (RR, 1.07; 95% bCrI, 1.01-1.14).

### Event Study Analyses

**Figure 2.**
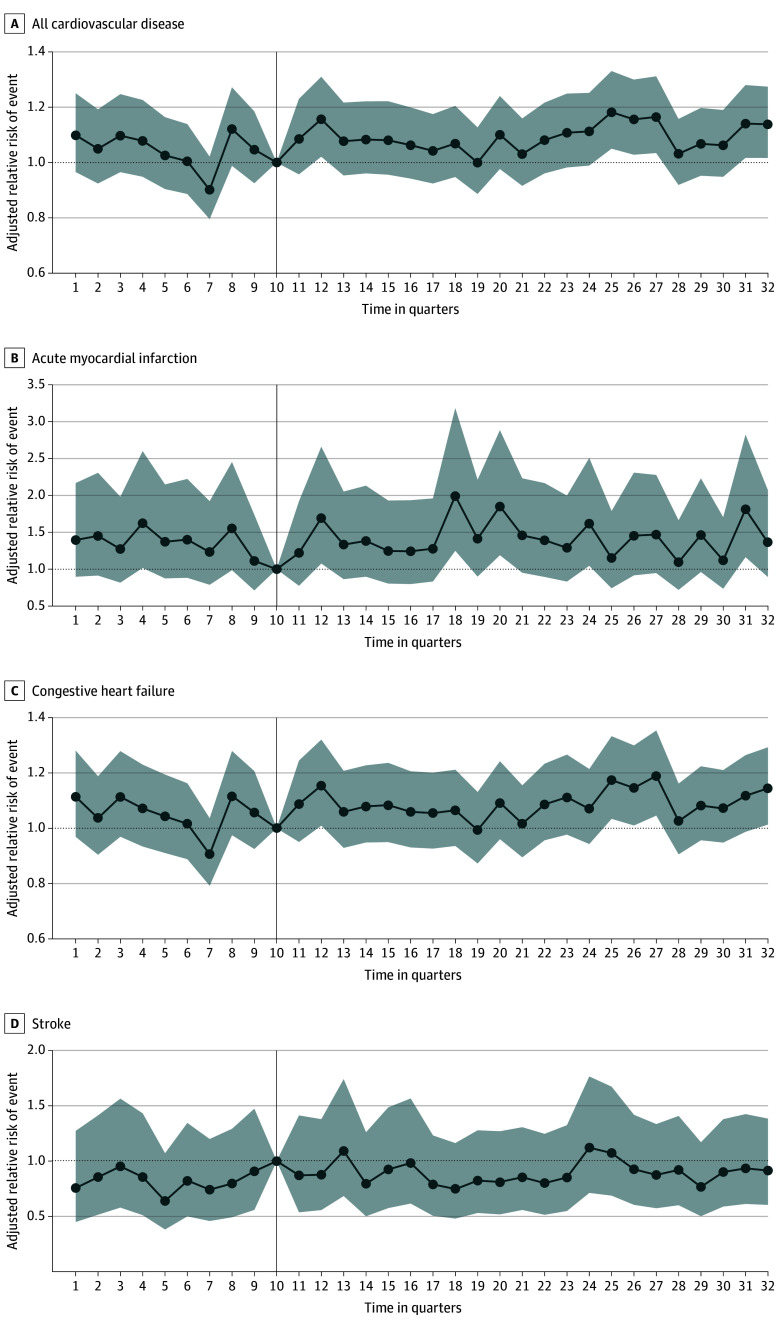
Adjusted Difference-in-Differences Estimates in the Association of Cardiovascular Disease (CVD)− and CVD Subgroup−Related Events With Hurricane Sandy Flood-Impact Exposure Across All Regions Quarter 2 represents Hurricane Sandy flood impact in 2012. Exposure regions include New Jersey, New York City, and Connecticut. Difference-in-differences estimates were calculated per 10 000 Medicare fee-for-service (FFS) beneficiaries. The event study model adjusted for zip code tabulation area (ZCTA)−level covariates calculated from the American Community Survey 5-year estimates (proportion of White residents, Area Deprivation Index, proportion of residents in ZCTA 65 years or older) and ZCTA-level covariates calculated from Medicare FFS beneficiaries (mean age, proportion of racial and ethnic minority residents, mean adjusted Charlson Comorbidity Index score); models included a spatiotemporal random effect with the temporal component modeled with a random walk and the spatial random effect defined by the Besag-York-Mollié model that smooths data according to neighborhood structure and uncorrelated spatial noise. The vertical line represents the reference.

Figure 2 describes a times series sequence of adjusted CVD and CVD subtype event rates for all regions in relation to the time quarter prior to Hurricane Sandy’s landfall (second quarter of 2012). In relation to the time quarter prior to Sandy’s landfall, we found that hurricane-related flooding was associated with an elevated risk of adjusted CVD event rates (and adjusted HF rates) 6 months later, and again with elevated risks 4 years after landfall (time quarters 25-27). When analyzed by each CVD subtype, other differences were found. While hurricane-related flooding was also associated with increased adjusted AMI rates 6 months after landfall, there were further increases 2 years (time quarters 18 and 20) and 5 years (time quarter 31) after landfall. We did not find any elevated adjusted risk of stroke or HF rates in the event study analysis across the study period.

### Sensitivity Analyses

Compared with findings from our primary analysis, findings that used generalized linear models with ordinary least squares regression were similar, with larger coefficients (eTable 3 in Supplement 1). Results and parameters for a model with 3:1 matching were comparable with those for 1:1 and 2:1 matching.

## Discussion

In this spatiotemporal econometric cohort study of Medicare FFS beneficiaries, we found that flooding from Hurricane Sandy was associated with an increased risk of CVD events as long as 5 years after hurricane landfall across all studied regions. In subgroup analyses, hurricane-related flooding was associated with increased adjusted CVD rates in New Jersey attributable to adjusted HF rates but not adjusted AMI nor stroke rates. In the event study analyses, hurricane-related flooding was associated with increased adjusted CVD, HF, and AMI rates 6 months after landfall, but we also observed elevated adjusted AMI rates 2 years after landfall and HF rates 4 to 5 years after landfall.

To our knowledge, this is the first study to examine, identify, and quantify the association between long-term CVD event risk in older populations and hurricane-related events. Our findings are consistent with previous studies that demonstrated short-term increased risk of AMI after Hurricane Sandy in New Jersey^41^ and hurricanes in general.^10,27^ While a series of studies also identified associations with a 2- to 4-fold increased rate of MI in single sites after Hurricane Katrina, including many years after landfall,^4^ others have rigorously shown increased 3% to 5% risk of all-cause mortality^42^ and 2.5% increased risk of CVD-related mortality^43^ associated with hurricane exposure as long as 2 years later. Our analysis suggests hurricane-related flooding may be associated with an increased risk of HF in the longer term (although regional variation exists) and increased risk of AMI events, with effect sizes similar to estimated mortality rates.

Our approach of using the difference-in-differences and event study methods served different but complementary purposes.^44^ The adjusted CVD event rates calculated using the difference-in-differences approach quantified the association between hurricane-related flooding and mean CVD (and subtype) event rates calculated throughout the entire posthurricane period (2013 to 2017). The event study approach allowed the quantification of the same association in time quarters in relation to the quarter prior to Hurricane Sandy’s landfall. Thus, while increased adjusted AMI and HF rates were found in the event study within 6 months of the hurricane’s landfall, when the mean was calculated over the entire posthurricane period, adjusted HF rates remained elevated. One possible reason is that within the Medicare population 65 years or older, the prevalence of HF is high compared with that of the general population and has increased over time.^45^

Our findings, although specific to Hurricane Sandy’s impact in NYC, New Jersey, and Connecticut, are nonetheless broadly relevant. Hurricane Sandy was one of the costliest hurricanes to make landfall in the US, owing in part to its impact on heavily urbanized areas along the East Coast.^46^ Moreover, our findings from the difference-in-differences analysis suggest an increased HF risk, particularly in New Jersey, raising not only the risk of future HF (and possibly AMI) events but also likely increasing associated health care costs through emergency department visits and inpatient stays. These findings have the potential to inform changes to existing disaster management frameworks to consider and address long-term effects of hurricane exposure beyond simply the prioritization of emergency care in the aftermath of hurricane events.

### Limitations

Our study has some limitations. First, our analysis considered only Medicare FFS beneficiaries who remained in the same ZCTA during the entire study period. This was done to ensure the stable unit treatment assumption of the difference-in-differences model specification was met.^47^ Previous work on disaster relocation has noted a protective effect against stress,^48,49^ a possible mechanism leading to CVD events in the postdisaster period, particularly for individuals with the financial resources to successfully relocate. Thus, it is possible that our findings may overestimate the CVD risk among all flood-exposed beneficiaries (including those who left). To address this limitation, we performed propensity score matching on sociodemographic and clinical covariates, showing good balance using the standardized mean difference.

Second, the cohort consisted only of community-based Medicare FFS beneficiaries and did not include institutionalized Medicare FFS beneficiaries or beneficiaries enrolled in Medicare Advantage. Thus, our findings may not be generalizable to the broader Medicare population.

Third, our analysis was performed at the ZCTA level, the smallest available spatial unit from Medicare claims data. Future work should consider undertaking individual-level beneficiary analysis.

Last, analysis was ecological in nature, with concern that the ecological fallacy may hold.^50^ However, our findings demonstrated important longer-term geographical variation in associated CVD risk. Thus, our findings underscore the importance of ecological analyses in analyzing risks associated with extreme weather events such as hurricanes, where hurricane impacts in both the short and the long term have wide-ranging effects across space and time.

## Conclusions

In this cohort study of Hurricane Sandy’s association with long-term CVD risk among Medicare FFS beneficiaries, hurricane-related flooding was associated with increases in CVD event rates as long as 5 years after landfall, largely attributable to increased adjusted HF rates in New Jersey. These findings highlight the importance of place-based vulnerability from hurricane exposure to mitigate CVD risk in the longer term and the need to consider long-term outcomes in hurricane mitigation efforts.

## Notes

Supplement 1. **eFigure 1.** Exclusion Cascade **eTable 1.** *International Classification of Disease, Ninth Revision* (*ICD-9*), and *International Statistical Classification of Disease, Tenth Revision* (*ICD-10*), Codes for Heart Failure, Stroke, and Acute Myocardial Infarction **eFigure 2.** Directed Acyclic Graph Informing Epidemiological Model **eTable 2.** Calculation of the Moran *I* Statistic and Associated *P* Value for CVD Outcome for All Regions During the Study Period (2010 to 2017) **eTable 3.** Adjusted Difference in Outcome Rates From 2 Years Before to 5 Years After Superstorm Sandy Landfall Between Flood-Impacted and Nonimpacted ZCTAs by Region, 2011 to 2017 **eFigure 3.** Diagnostics and Outcomes of Matching **eFigure 4.** Model Specification and Estimating Equations for Difference-in-Differences and Event Study Framework

Supplement 2.
Data Sharing Statement
