# Supplementary material for: Hurricane Exposure and Risk of Long-Term Cardiovascular Disease Outcomes
Source: JAMA Netw Open. 2025 Sep 3;8(9):e2530335. doi: 10.1001/jamanetworkopen.2025.30335 (PMC12409596; doi:10.1001/jamanetworkopen.2025.30335)
Supplement: Supplement 1. — eFigure 1. Exclusion Cascade eTable 1. International Classification of Disease, Ninth Revision (ICD-9), and International Statistical Classification of Disease, Tenth Revision (ICD-10), Codes for Heart Failure, Stroke, and Acute Myocardial Infarction eFigure 2. Directed Acyclic Graph Informing Epidemiological Model eTable 2. Calculation of the Moran I Statistic and Associated P Value for CVD Outcome for All Regions During the Study Period (2010 to 2017) eTable 3. Adjusted Difference in Outcome Rates From 2 Years Before to 5 Years After Superstorm Sandy Landfall Between Flood-Impacted and Nonimpacted ZCTAs by Region, 2011 to 2017 eFigure 3. Diagnostics and Outcomes of Matching eFigure 4. Model Specification and Estimating Equations for Difference-in-Differences and Event Study Framework [file jamanetwopen-e2530335-s001.pdf]

Supplementary Online Content

Ghosh AK, Soroka O, Safford M, et al. Hurricane exposure and risk of long-term cardiovascular disease outcomes. *JAMA Netw Open.* 2025;8(9):e2530335. doi:10.1001/jamanetworkopen.2025.30335

eFigure 1. Exclusion Cascade
eTable 1. *International Classification of Disease, Ninth Revision* (*ICD-9*), and *International Statistical Classification of Disease, Tenth Revision* (*ICD-10*), Codes for Heart Failure, Stroke, and Acute Myocardial Infarction
eFigure 2. Directed Acyclic Graph Informing Epidemiological Model
eTable 2. Calculation of the Moran *I* Statistic and Associated *P* Value for CVD Outcome for All Regions During the Study Period (2010 to 2017)
eTable 3. Adjusted Difference in Outcome Rates From 2 Years Before to 5 Years After Superstorm Sandy Landfall Between Flood-Impacted and Nonimpacted ZCTAs by Region, 2011 to 2017
eFigure 3. Diagnostics and Outcomes of Matching
eFigure 4. Model Specification and Estimating Equations for Difference-in-Differences and Event Study Framework

This supplementary material has been provided by the authors to give readers additional information about their work.

© 2025 Ghosh AK et al. *JAMA Network Open*.

eFigure 1. Exclusion Cascade

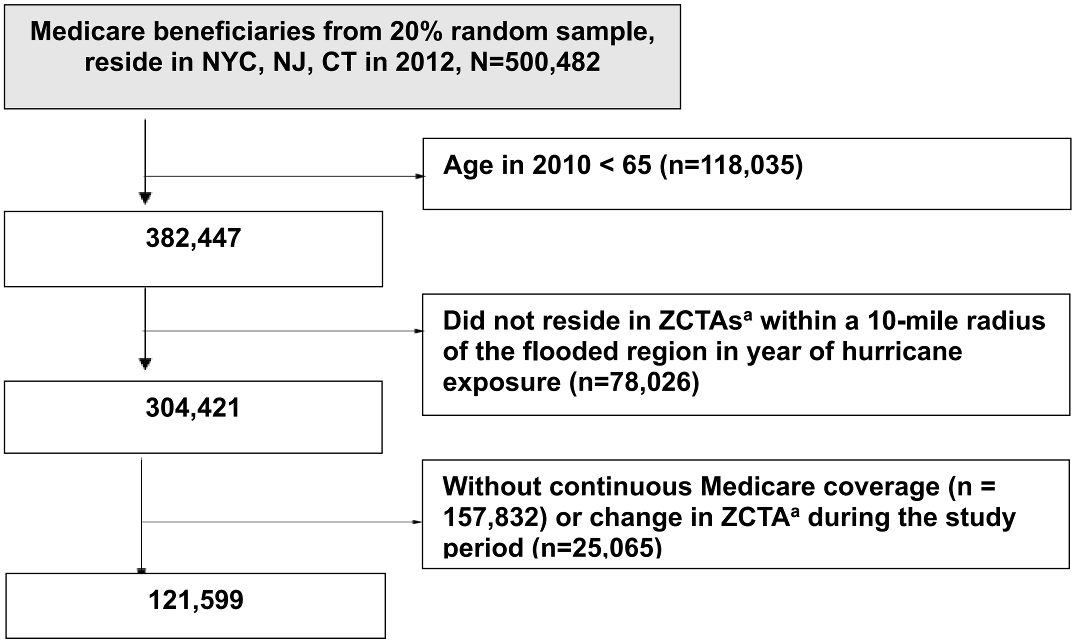

a ZCTA: ZIP code tabulation area.

© 2025 Ghosh AK et al. *JAMA Network Open*.

eTable 1. *International Classification of Disease, Ninth Revision* (*ICD-9*), and *International Statistical Classification of Disease, Tenth Revision* (*ICD-10*), Codes for Heart Failure, Stroke, and Acute Myocardial Infarction

**Condition**	**ICD-9 codes**	**ICD-10 codes**
Acute myocardial infarction (AMI)	AMI was defined using at least 1 inpatient claim with 410.01, 410.11, 410.21, 410.31, 410.41, 410.51, 410.61, 410.71, 410.81, 410.91, 411.1 (any DX on the claim).	AMI was defined using at least 1 inpatient claim with: I21.01, I21.02, I21.09, I21.11, I21.19, I21.21, I21.29, I21.3, I21.4, I21.9, I21.A1, I21.A9, I21.B (any DX on the claim).
Stroke[a]	430, 431, 433.01, 433.11, 433.21, 433.31, 433.81, 433.91, 434.01, 434.11, 434.91, 436	I60.xx, I61.xx, I63.xx
Heart failure[c]	HF was defined using at least 1 inpatient claim or at least 2 outpatient claims (at least 30 days apart): 398.91, 402.01, 402.11, 402.91, 404.01, 404.03, 404.11, 404.13, 404.91, 404.93, 428.0, 428.1, 428.20, 428.21, 428.22, 428.23, 428.30, 428.31, 428.32, 428.33, 428.40, 428.41, 428.42, 428.43, 428.9	HF was defined using at least 1 inpatient claim or at least 2 outpatient claims (at least 30 days apart): I09.81, I11.0, I13.0, I13.2, I50.1, I50.20, I50.21, I50.22, I50.23, I50.30, I50.31, I50.32, I50.33, I50.40, I50.41, I50.42, I50.43, I50.810, I50.811, I50.812, I50.813, I50.814, I50.82, I50.83, I50.84, I50.89, I50.9.

a. If any of the qualifying claims have injury diagnosis (ICD9 800 <= DX Code <= 804.99, 850 <= DX Code <= 854.19; ICD10 S0190XA, S02xx, S06.xx [Stroke CCW definition] in any DX position OR DX ICD9 V57xx, ICD10 Z51.89 as the principal DX Code), then claim was excluded from Stroke claims.
b. Only first or second DX on the inpatient or outpatient institutional claim, line diagnosis on the outpatient non-institutional claim
c. To ensure that service was provided by licensed health care professional, all HF outpatient claims were subset to face-to-face, Emergency department [ED], urgent care [UC], observation [Obs], critical care (CC) visits: institutional – using Healthcare Common Procedure Coding System (HCPCS), revenue center codes; non-institutional – using HCPCS, place of service codes criteria.

© 2025 Ghosh AK et al. *JAMA Network Open*.

eFigure 2. Directed Acyclic Graph Informing Epidemiological Model

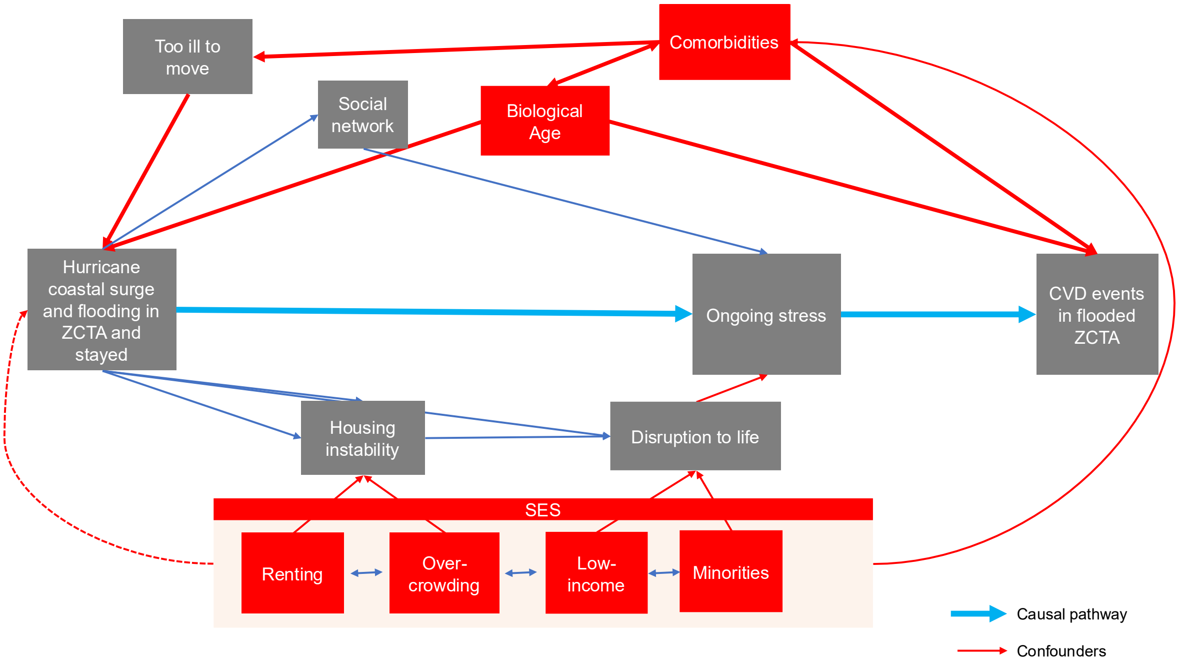

© 2025 Ghosh AK et al. *JAMA Network Open*.

eTable 2. Calculation of the Moran *I* Statistic and Associated *P* Value for CVD Outcome for All Regions During the Study Period (2010 to 2017)

Year	Quarter	Moran's I	P-value
2010	First	0.503	<0.001
2010	Second	0.501	<0.001
2010	Third	0.503	<0.001
2010	Fourth	0.517	<0.001
2011	First	0.459	<0.001
2011	Second	0.518	<0.001
2011	Third	0.419	<0.001
2011	Fourth	0.505	<0.001
2012	First	0.491	<0.001
2012	Second	0.501	<0.001
2012	Third	0.526	<0.001
2012	Fourth	0.506	<0.001
2013	First	0.503	<0.001
2013	Second	0.509	<0.001
2013	Third	0.520	<0.001
2013	Fourth	0.540	<0.001
2014	First	0.490	<0.001
2014	Second	0.484	<0.001
2014	Third	0.450	<0.001
2014	Fourth	0.434	<0.001
2015	First	0.485	<0.001
2015	Second	0.466	<0.001
2015	Third	0.494	<0.001
2015	Fourth	0.501	<0.001
2016	First	0.493	<0.001
2016	Second	0.465	<0.001
2016	Third	0.438	<0.001
2016	Fourth	0.449	<0.001
2017	First	0.448	<0.001
2017	Second	0.460	<0.001
2017	Third	0.437	<0.001
2017	Fourth	0.471	<0.001

© 2025 Ghosh AK et al. *JAMA Network Open*.

eTable 3. Adjusted Difference in Outcome Rates From 2 Years Before to 5 Years After Superstorm Sandy Landfall Between Flood-Impacted and Nonimpacted ZCTAs by Region, 2011 to 2017

	All Regions	NYC	NJ	CT
	**Cardiovascular Disease (95% confidence intervals)**			
All ZCTAs	1.01 (0.97-1.05)	1.02 (0.95-1.09)	1.07 (1.01-1.13)*	0.96 (0.85-1.10)
ZCTAs ≥ 50 beneficiaries	1.02 (0.98-1.06)	1.02 (0.96-1.09)	1.08 (1.02-1.14)*	1.00 (0.87-1.14)
ZCTAs ≥ 100 beneficiaries	1.02 (0.98-1.07)	1.02 (0.95-1.09)	1.10 (1.04-1.17)*	0.97 (0.83-1.13)
	**Myocardial Infarction (95% confidence intervals)**			
All ZCTAs	1.05 (0.93-1.19)	0.93 (0.75-1.16)	1.17 (1.00-1.36)*	0.91 (0.6-1.36)
ZCTAs ≥ 50 beneficiaries	1.05 (0.93-1.19)	0.93 (0.75-1.16)	1.16 (0.98-1.36)	0.95 (0.63-1.44)
ZCTAs ≥ 100 beneficiaries	1.04 (0.91-1.19)	0.92 (0.73-1.15)	1.13 (0.95-1.34)	1.00 (0.62-1.57)
	**Heart Failure (95% confidence intervals)**			
All ZCTAs	1.00 (0.96-1.05)	1.02 (0.96-1.10)	1.06 (1.00-1.13)*	0.94 (0.81-1.08)
ZCTAs ≥ 50 beneficiaries	1.01 (0.96-1.06)	1.03 (0.96-1.10)	1.06 (1.00-1.13)*	0.97 (0.84-1.13)
ZCTAs ≥ 100 beneficiaries	1.01 (0.97-1.07)	1.03 (0.96-1.10)	1.10 (1.02-1.17)*	0.91 (0.77-1.08)
	**Stroke (95% confidence intervals)**			
All ZCTAs	1.06 (0.93-1.21)	0.91 (0.70-1.16)	1.08 (0.92-1.27)	1.29 (0.85-1.94)
ZCTAs ≥ 50 beneficiaries	1.09 (0.95-1.24)	0.88 (0.68-1.13)	1.13 (0.95-1.33)	1.38 (0.89-2.10)
ZCTAs ≥ 100 beneficiaries	1.11 (0.97-1.28)	0.90 (0.69-1.16)	1.15 (0.96-1.37)	1.55 (0.94-2.52)

1 All models utilized negative binomial-distributed log-link function and were adjusted for ZCTA-level covariates calculated from the American Community Survey 5-year estimates (proportion White, Area Deprivation Index, proportion of residents in ZCTA aged 65 years and older) and ZCTA-level covariates calculated from Medicare FFS beneficiaries datasets (mean age, average adjusted Charlson score)
* $p < 0.001$
ZCTA is ZIP Code Tabulation Area

© 2025 Ghosh AK et al. *JAMA Network Open*.

eFigure 3. Diagnostics and Outcomes of Matching

A) Nearest neighbor matching 1:1 with replacement

i) Distribution of Propensity Scores

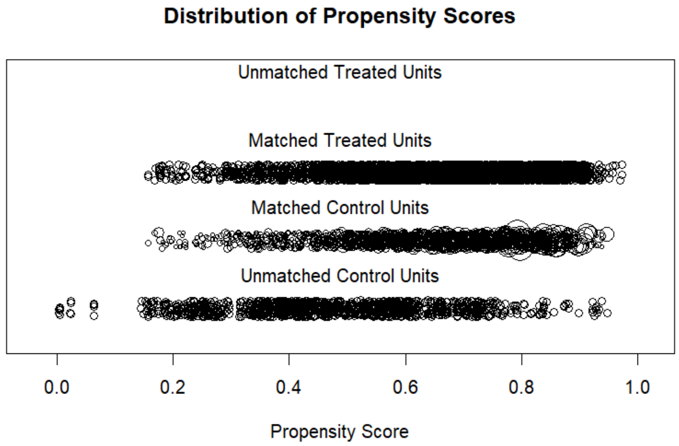

ii) Table of values post-matching, with standard mean difference

Covariate	Means Treated	Means Control	Std. Mean Diff.
Distance	0.6855	0.6855	0.0002
Mean Age	79.7476	79.9659	-0.1566
Fraction over 65 years	0.1448	0.1519	-0.1048
Fraction residing in rent occupied household	0.4048	0.3979	0.0266
Average adjusted Charlson score	0.8377	0.8801	-0.1583
ADI, National rank	23.9471	25.6369	-0.0993
Fraction of females by ZCTA	0.613	0.6218	-0.1329
Fraction of overcrowded households by ZCTA	0.0386	0.0403	-0.0349
Median household income, normalized	-0.3552	-0.3979	0.0506
Fraction of racial minorities	0.2995	0.3056	-0.0246
Region CT	0.161	0.1846	-0.0642
Region NJ	0.5624	0.5281	0.069
Region NYC	0.2766	0.2873	-0.0238

iii) Table of values post-matching, with standard mean difference

	Cardiovascular Disease (95% Bayesian CI)
All ZCTA	1.05 (1.01 to 1.08)
ZCTAs ≥ 50 beneficiaries	1.06 (1.02 to 1.09)
ZCTAs ≥ 50 beneficiaries	1.07 (1.03 to 1.11)

B) Nearest neighbor matching 2:1 with replacement

© 2025 Ghosh AK et al. *JAMA Network Open*.

i) Distribution of Propensity Scores

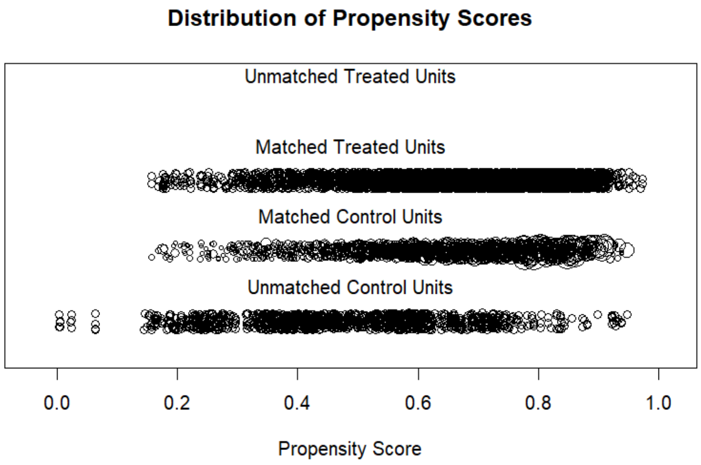

ii) Table of values post-matching, with standard mean difference

**Covariate**	**Means Treated**	**Means Control**	**Std. Mean Diff.**
Distance	0.6855	0.6855	0.0003
Mean Age	79.7476	79.8772	-0.0931
Fraction over 65 years	0.1448	0.1496	-0.0710
Fraction residing in rent occupied household	0.4048	0.3939	0.042
Average adjusted Charlson score	0.8377	0.8683	-0.1144
ADI, National rank	23.9471	25.3342	-0.0815
Fraction of females by ZCTA	0.613	0.6201	-0.1079
Fraction of overcrowded households by ZCTA	0.0386	0.0395	-0.0187
Median household income, normalized	-0.3552	-0.3848	0.0350
Fraction of racial minorities	0.2995	0.2993	0.0009
Region CT	0.1610	0.1879	-0.0731
Region NJ	0.5624	0.5624	0.5342
Region NYC	0.2766	0.2766	0.2779

iii) DiD estimators

	Cardiovascular Disease (95% Bayesian CI)
All ZCTA	1.05 (1.01 to 1.09)
ZCTAs ≥ 50 beneficiaries	1.06 (1.02 to 1.09)
ZCTAs ≥ 50 beneficiaries	1.07 (1.03 to 1.12)

C) Nearest neighbor matching 3:1 with replacement

i) Distribution of Propensity Scores

© 2025 Ghosh AK et al. *JAMA Network Open*.

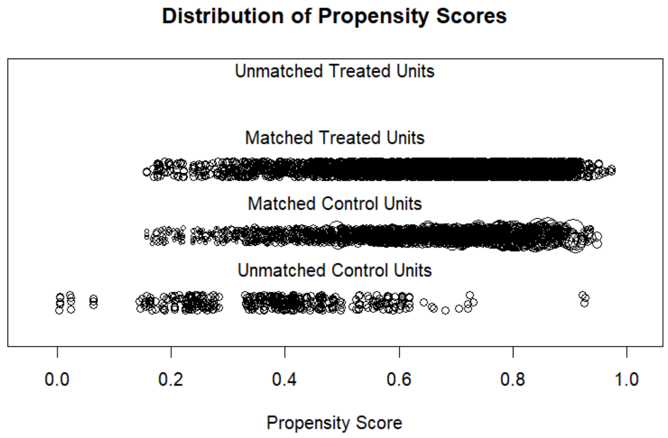

ii) Table of values post-matching, with standard mean difference

Covariate	Means Treated	Means Control	Std. Mean Diff.
Distance	0.6855	0.6855	0.0003
Mean Age	79.7476	79.8511	-0.0743
Fraction over 65 years	0.1448	0.15	-0.0776
Fraction residing in rent occupied household	0.4048	0.3944	0.0399
Average adjusted Charlson score	0.8377	0.8594	-0.0813
ADI, National rank	23.9471	25.3819	-0.0843
Fraction of females by ZCTA	0.613	0.62	-0.1052
Fraction of overcrowded households by ZCTA	0.0386	0.0398	-0.0261
Median household income, normalized	-0.3552	-0.3901	0.0413
Fraction of racial minorities	0.2995	0.299	0.0023
Region CT	0.161	0.1861	-0.0683
Region NJ	0.5624	0.5407	0.0436
Region NYC	0.2766	0.2732	0.0078

iii) DiD estimators for primary analysis

	Cardiovascular Disease (95% Bayesian CI)
All ZCTA	1.05 (1.01 to 1.08)
ZCTAs ≥ 50 beneficiaries	1.06 (1.02 to 1.09)
ZCTAs ≥ 50 beneficiaries	1.07 (1.03 to 1.11)

© 2025 Ghosh AK et al. *JAMA Network Open*.

eFigure 4. Model Specification and Estimating Equations for Difference-in-Differences and Event Study Framework

The generalized linear model employed for the difference-in-differences model was:

$$\mathrm{E}\left(\log\left(1000 \times \frac{Y_{it}}{\mathrm{Population}_{it}}\right)\right) = \beta_0 + \beta \ \ (Flood_i \times Posthurricane_t) + \mu_{it} + \nu_i + \theta_k + \varepsilon_{ijt}$$

The generalized linear model for the event study model was:

$$\mathrm{E}\left(\log\left(1000 \times \frac{Y_{it}}{\mathrm{Population}_{it}}\right)\right) = \beta_0 + \sum_{j=-9}^{22} \beta_j \ (\mathrm{Time}_j \times Flood_i) + \mu_{it} + \nu_i + \theta_k + \varepsilon_{ijt}$$

where:

- Y_{it} denotes the number of CVD events in ZCTA i in quarter t, where t ranges from -9 (9 quarters before hurricane impact) to 22 (22 quarters after hurricane impact), where t = 0 is the second quarter of 2012 (one quarter before Hurricane Sandy struck)
- Time_j is a binary variable indicating whether or not $j = t$. Time $t = 0$ (the quarter prior to the hurricane's landfall is treated as the reference level.
- $Flood$ is an indicator variable indicating whether or not the ZCTA$_i$ was flooded from Hurricane Sandy.
- $Posthurricane_t$ is a indicator variable of the time period after Hurricane Sandy struck
- The quarter-specific coefficients $\beta_{j,\mathrm{i}}$ can be interpreted as the difference in log CVD rates in flooded ZCTAs at time j, relative to time 0. When exponentiated, they are interpreted as multiplicative factors rather than differences.
- μ_{it} denotes yearly ZCTA-level fixed effects, including the effect of the ZCTA's median income, percentage of residents who are white, and percentage of residents below the federal poverty threshold.
- ν_i denotes a spatial random effect term for ZCTA i. In particular, it is a conditionally autoregressive term with a Besag-York-Mollié specification; this means that conditioned on the spatial random effect terms for all other ZCTAs, ν_i is modeled as normally-distributed with mean $\frac{1}{|N_i|}\sum_{j \in N_i} \nu_j$, where N_i are the indices of the immediately-neighboring ZCTAs.
- θ_k denotes yearly time fixed effects where k ranges from -2 (2 years prior to hurricane exposure) to 5 (5 years after hurricane exposure)
- $\mathrm{Population}_{it}$ is the population of ZCTA i in year t.

© 2025 Ghosh AK et al. *JAMA Network Open*.

© 2025 Ghosh AK et al. *JAMA Network Open*.
