# Supplementary material for: Hurricane Exposure and Risk of Long-Term Cardiovascular Disease Outcomes
Source: JAMA Netw Open. 2025 Sep 3;8(9):e2530335. doi: 10.1001/jamanetworkopen.2025.30335 (PMC12409596; doi:10.1001/jamanetworkopen.2025.30335)
Supplement: Supplement 2. — Data Sharing Statement [file jamanetwopen-e2530335-s002.pdf]

Data Sharing Statement

Ghosh. Hurricane Exposure and Risk of Long-Term Cardiovascular Disease Outcomes. *JAMA Netw Open*. Published September 03, 2025. doi:10.1001/jamanetworkopen.2025.30335

Data
Data available: No

Additional Information
Explanation for why data not available: Taking Medicare claims
